# Development of a new hybrid approach combining AFM and SEM for the nanoparticle dimensional metrology

**DOI:** 10.3762/bjnano.10.150

**Published:** 2019-07-26

**Authors:** Loïc Crouzier, Alexandra Delvallée, Sébastien Ducourtieux, Laurent Devoille, Guillaume Noircler, Christian Ulysse, Olivier Taché, Elodie Barruet, Christophe Tromas, Nicolas Feltin

**Affiliations:** 1 Laboratoire National de métrologie et d’Essais – Nanometrology, 29 avenue Hennequin, 78197 Trappes Cedex, France; 2 Institut Prime Département Physique et Mécanique des Matériaux, 11 Bd Marie et Pierre Curie, 86962 Futuroscope Chasseneuil, France; 3 Centre de Nanosciences et de Nanotechnologies C2N, route de Nozay, 91460 Marcoussis, France; 4 LIONS, NIMBE, CEA, CNRS, Université Paris Saclay, CEA Saclay, 91191 Gif-sur-Yvette, France

**Keywords:** AFM, hybrid metrology, nanoparticles, SEM, size distribution, uncertainty budget

## Abstract

At this time, there is no instrument capable of measuring a nano-object along the three spatial dimensions with a controlled uncertainty. The combination of several instruments is thus necessary to metrologically characterize the dimensional properties of a nano-object. This paper proposes a new approach of hybrid metrology taking advantage of the complementary nature of atomic force microscopy (AFM) and scanning electron microscopy (SEM) techniques for measuring the main characteristic parameters of nanoparticle (NP) dimensions in 3D. The NP area equivalent, the minimal and the maximal Feret diameters are determined by SEM and the NP height is measured by AFM. In this context, a kind of new NP repositioning system consisting of a lithographed silicon substrate has been specifically developed. This device makes it possible to combine AFM and SEM size measurements performed exactly on the same set of NPs. In order to establish the proof-of-concept of this approach and assess the performance of both instruments, measurements were carried out on several samples of spherical silica NP populations ranging from 5 to 110 nm. The spherical nature of silica NPs imposes naturally the equality between their height and their lateral diameters. However, discrepancies between AFM and SEM measurements have been observed, showing significant deviation from sphericity as a function of the nanoparticle size.

## Introduction

AFM (atomic force microscopy) or SEM (scanning electron microscopy) are considered to be reference techniques for measuring the size of nanoparticles (NPs) because the measurements are based on a direct observation of the imaged NP population. This creates a direct link between the NP dimensional measurement and the meter definition in the international system (SI) of units [1]. The involved measurand is then a geometrical size. Unlike microscopy-based techniques, all other NP sizing techniques, for instance, dynamic light scattering, centrifugal liquid sedimentation, and particle tracking analysis [2] are classified as indirect because the size measurement is the result of a calculation or a modelling process.

However, although AFM and SEM are direct techniques and make it possible to obtain the geometrical size of NPs, their measuring principles are very different and the measurands defined for both techniques are distinct.

AFM is a technique used for mapping physical properties at the nanoscale. The measuring principle is based on the detection of the interaction (attractive or repulsive forces) between a sharp tip attached to the end of a flexible cantilever and a sample. In contact mode, the interaction force is kept constant during the scanning thanks to a feedback loop that controls the tip–sample distance. This mode is not really suitable for NP imaging because the NPs might be displaced by the tip over the sample. To avoid this effect, the intermittent contact (tapping) mode is more commonly used. It consists in oscillating the cantilever near its resonant frequency and maintaining a constant amplitude during the scan. However, regardless of the used mode, and due to tip convolution, the obtained image is a function of tip shape and tip radius (estimated to be around a dozen of nanometers) [3–4].

As a matter of fact, the value of the tip radius gets comparable to the NP size. This induces a non-negligible broadening of the measured NP lateral dimensions and can seriously affect the measurement in the *XY*-plane. However, concerning spherical nanoparticles, AFM can be used for an accurate determination of the NP diameter by just measuring their heights because the convolution has no effect on the measurement of the highest point of the NP [1].

In SEM, an electron beam scans the sample and several interactions can occur between the incident electrons and the atoms of the sample surface. Inelastic interactions lead to the creation of secondary electrons (SEs) that may exit to the sample and be collected by a specific detector. An image of the surface is then constructed based on the number of SEs collected for each pixel during scanning. Thus, the *Z*-axis data are only related to signal intensity (greyscale) giving no metrological information. Consequently, NP dimensions extracted from SEM images contain only the dimensions in the *XY*-plane. Generally, the measurand used for the described NP size in SEM is an area-equivalent diameter. This corresponds to the diameter of a sphere with the same projected surface as the studied nano-object. However, from this imaged NP surface area, other measurands such as minimum and maximum Feret diameters can be defined as well [5].

As a result, SEM and AFM are complementary. Indeed, SEM gives no quantitative information about the NP height, whereas the uncertainty associated with the AFM measurement of the NP maximum point is close to 1.5 nm [1]. Conversely, the lateral dimensions measured by AFM are impacted by tip/NP convolution, whereas latest-generation scanning electron microscopes equipped with field-emission guns can reach a resolution of 1 nm in the *XY*-plane.

Consequently, we propose in this paper the development of a hybrid metrology that allows for the measurement of the characteristic dimensions of a nano-object in 3D, by combining the measurements performed with AFM and SEM. The concept of hybrid metrology has been recently defined in the semiconductor industry [6–8]. In fact, the challenges posed by the constraints of Moore’s law with a continuous shrinkage of transistor dimensions are huge in terms of metrology. The abandonment of a technology based only on silicon and the emergence of 3D architectures for microprocessors of future generations will induce a genuine revolution in the measuring techniques.

This concept assumes that a single technique alone cannot meet the metrological needs required to support the development of technology nodes below 22 nm [9]. Thus, hybrid metrology consists in using several measuring techniques and their associated metrologies to combine their strengths and limit their weaknesses. Combined with statistical tools and input data, such as chemical information and crystal structure, hybrid metrology makes it possible, after data fusion, to obtain more reliable measurements and uncertainties better than the uncertainties associated with the measurements provided by each instrument [10].

A previous study had been performed on the comparison of AFM and SEM measurements on the same set of nanoparticles [11]. This study especially proposed a robust Matlab routine for data processing from AFM and SEM measurements on nanoparticles. The comparison between both techniques had been carried out on a single silica NP population with few NPs measured. In this new paper, we propose to extend this strategy by combining AFM and SEM measurements for several silica NP populations. Moreover, additional tools such as calibration standard and repositioning system have been developed in this study to easily find the area of interest.

However, the proof of concept of this method requires three steps. Firstly, the development of a specific reference structure (standard) suitable for investigating NPs is required. Actually, the calibration certificates associated with reference structures commercially available do not give values within 1 nm along the *XYZ*-axes. Our new standard will make it possible to establish a “traceability route” between both instruments. The calibration grating is used for the comparison of the measurements performed by AFM and SEM and these measurements are traceable to SI units with the calibration of this standard through the implementation of the metrological AFM (mAFM) of LNE [12]. Secondly, we have to be capable of measuring exactly the same set of nanoparticles with both techniques. This implies that we have to relocalize the same area of interest (typically 5 μm × 5 μm) after moving the sample from AFM to SEM. The development of a repositioning system is then necessary. Finally, in order to investigate the limits of this method, the measurement of a population of spherical NPs is performed. We used silica (SiO_2_) nanoparticles that are supposed to have a spherical shape [13–14]. Indeed, the sphericity requires that the NP height is equal to the diameter measured in *XY*-plane (lateral diameters). In this manner, height measurements performed by AFM can be compared with diameter measurements (min Feret (*D*_Fmin_), max Feret (*D*_Fmax_) and area-equivalent diameter (*D*_SEM_)) performed by SEM.

## Experimental

### Materials and methods

Several samples of silica NP suspensions were investigated. ERM-FD102 and ERM-FD304 are certified reference samples provided by the Joint Research Centre Institute for Reference Material and Measurements (JRC-IRMM). For ERM-FD102, the certified size value for electron microscopy techniques (number-weighted modal area-equivalent diameter) is given to be 18.2 nm with an uncertainty of 1.6 nm (*k* = 2) for a first size class (called size class A) and 84 nm with a 2.1 nm uncertainty (*k* = 2) for a second size class (called size class B) [15]. Concerning ERM-FD304, the certification only covers dynamic light scattering and centrifugal liquid sedimentation as measuring methods. It also gives an indicative value for microscopy-based techniques (SEM and TEM) of 27.8 nm with 1.5 nm uncertainty (*k* = 2) [16]. The third sample, Klebosol^®^ 30R50, is a commercially available bimodal silica NP suspension. It is used for applications concerning catalysis, leather treatment, paints and coatings, and textiles. A complete dimensional characterization of this suspension has been carried out by using various techniques [17]. The last sample used, OT R3, is a single-mode silica suspension produced in a laboratory using the Stöber method [18]. The suspension has a nominal NP diameter of around 100 nm. Each silica NP suspension was diluted in water and deposited on silicon substrates through the spin-coating method detailed in [11]. This method yields well-dispersed nanoparticles on the substrate while maximizing the number of isolated NPs preventing agglomeration of the NPs.

NP SEM images have been recorded using a Zeiss ULTRA-Plus field-emission (FE) microscope equipped with a GEMINI optical column with an in-lens detector. All SEM measurements have been performed using the same adjustment parameters. The extra-high tension (EHT, accelerating voltage) corresponding to the incident electron energy at the time of interaction with sample is set at 3 kV. The working distance (WD), defined as being the distance between the sample and the bottom of the SEM column, corresponding also to the focal distance of the beam, is kept constant at 3 mm. According to the manufacturer specifications, the FE-SEM resolution is roughly 1.7 nm for EHT = 1 kV and 1.0 nm at 15 kV (for a working distance set at 2 mm). All others scan parameters, including pixel size, scan speed, contrast, and brightness, are fixed for all images. The pixel size was set to 1.4 nm with a total cycle time to record an image of 28.4 s. The values assigned for contrast and brightness were 31.4% and 49%, respectively.

The AFM measurements were carried out with a Veeco Nanoman V equipped with an accurate three-axis scanner operating under closed-loop control (hybrid *XYZ*-scanner with a range of 90 µm × 90 µm × 8 µm). All measurements were performed in air using tapping mode and OTESPA-R3 probes. The cantilever resonance frequency is 300 kHz and the nominal radius of curvature of the tip is roughly 7 nm. The nominal stiffness of the cantilever is 42 N/m. For all measurements, the tip oscillation amplitude was about 40 nm. The amplitude setpoint was fixed very high and near the free amplitude (80%) value to prevent too strong interactions with the sample and subsequent NP displacements. For vibration considerations, each instrument sits on a massive concrete block decoupled from the building. Furthermore, the AFM instrument is placed in an enclosure to protect it against acoustic noise and installed on an anti-vibration table. The pixel size was set to 5.0 nm for all AFM measurements. Moreover, the scanning parameters are fixed regardless of the NP population under study. The scan speed is equal to 4 µm/s with constant PID parameters set at 0.8 for integral gain and 10 for the proportional one.

Laboratory humidity and temperature are well controlled and stabilized (50 ± 5% relative humidity and 20 ± 0.15 °C for temperature). These precautions combined with the instrument performances make it possible to obtain low drifts and to carry out measurements with low noise.

### Basic measuring principle and data processing

**Figure 1:**
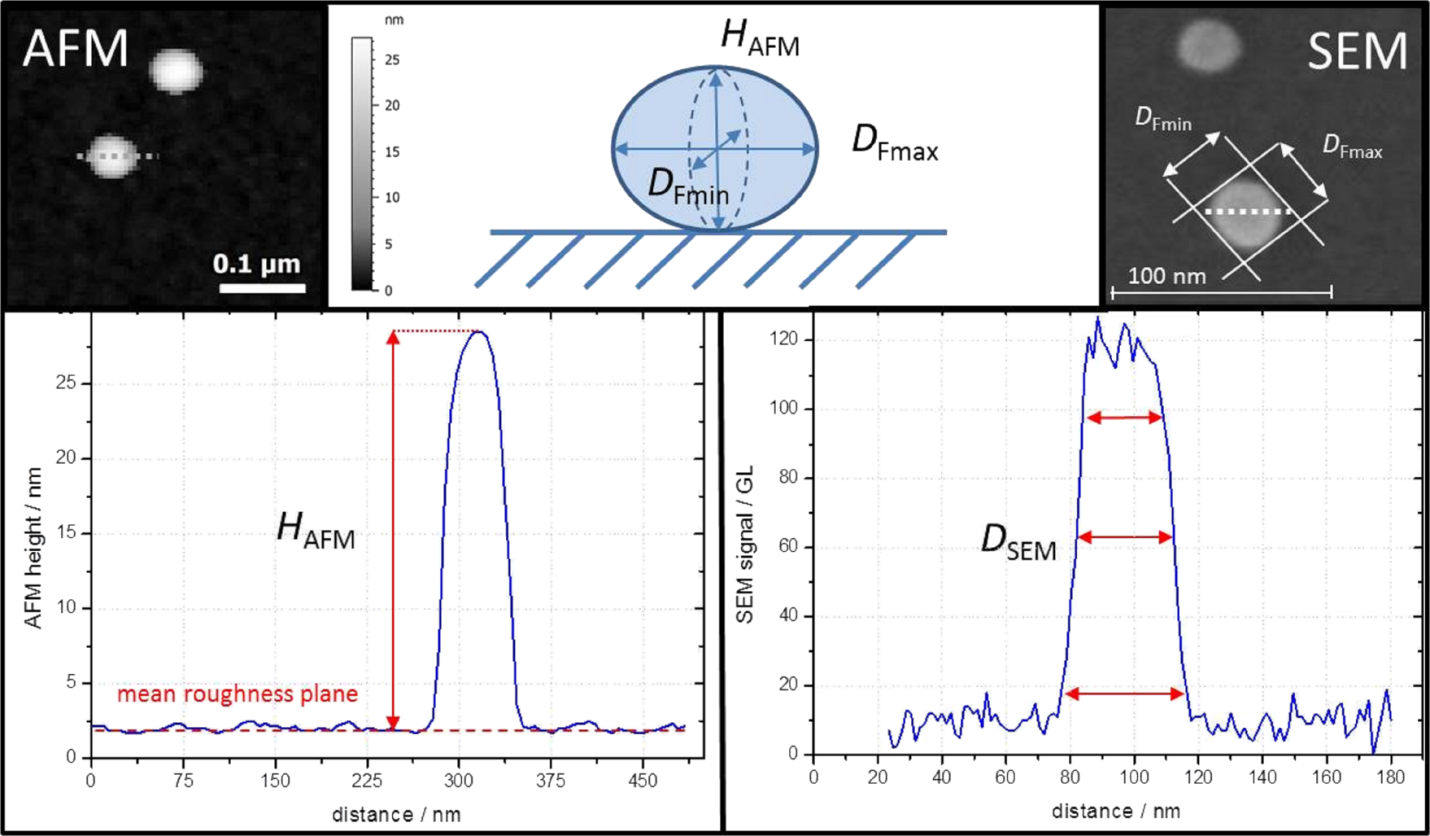
Basic principle for measuring the NP height by AFM and NP lateral diameters by SEM.

In this paper, the 3D characteristic dimensions of a NP silica population are defined through the NP height measured by AFM and their lateral diameters (min and max Feret diameters) measured by SEM. The spherical silica NP height, *H*_AFM_, was determined by subtracting the NP maximum point *Z*-coordinate measured by AFM from the position of the mean roughness plane as considered in [1]. The measuring principle is schematized in Figure 1. The roughness of the substrate surface impacts the uncertainty associated with the height measurement [1]. A silicon wafer has been chosen as substrate for NP deposition because its roughness is relatively low (*S*_q_ = 0.3 nm), its surface physicochemical features are particularly suitable for an optimized NP dispersion and its electrical properties are compatible with SEM measurements [11].

In the Figure 1 is also shown the principle implemented for measuring the lateral NP diameters in SEM. The min and max Feret diameters, *D*_Fmin_ and *D*_Fmax_, are determined from the projected image of the nanoparticle on *XY*-plane. Typically, the profile built from the SEM image of a single NP, as given in Figure 1, exhibits edges that are not completely vertical. Hence, one problem immediately arises: Where is the profile width to be measured to get a reliable measurement of the NP lateral diameter? In a previous study, by comparing the results experimentally obtained by AFM and SEM on nearly spherical silica NP, we have demonstrated that the method most consistent with AFM values was to take the full width at half maximum (FWHM) [19]. The profile FWHM depends on the accelerating voltage (or EHT) corresponding to the primary electron energy as shown in Figure 1. Above 4 kV, the signal-to-noise ratio, corresponding to the difference in grey level between the NP and the substrate, is too low. Hence, the profile widens with the accelerating voltage and uncertainty associated with the measurement increases.

The images were processed with a specific software developed in previous works and detailed in [1,11]. Concerning AFM, the approach consists in levelling the image, binarizing the image to discriminate objects from the substrate, identifying each imaged nano-object, evaluating roughness and building the size distribution histogram by only counting isolated nanoparticles. NP agglomeration may induce errors in the measurements and should be avoided [1,11]. The program is not able to distinguish agglomerates and isolated nanoparticles. But the operator can choose to include only the isolated NPs in the histogram through a bounding box. Another algorithm has been added to the software for the calculation of lateral diameters (*D*_Fmin_ and *D*_Fmax_) from SEM measurements [11].

### Precautions

Before starting the measurements, several precautions must be taken. During SEM imaging, molecules already existing at the sample surface desorb in the chamber vacuum or diffuse on the substrate and accumulate in the irradiated part of the sample [19] forming a contamination layer at the sample surface. This layer modifies the position of the reference plane during the AFM measurement. This is why it is necessary to start with the characterization of the NPs by AFM.

Moreover, a contamination layer could form on the substrate during drying of the suspension. In fact, the nanoparticles might be located not on the substrate surface but on a deformable contamination layer. This phenomenon has been observed in [19]. As a reminder, the height of a nanoparticle is calculated as the height maximum of the NP minus the mean surface height of the substrate. If this mean surface height cannot be clearly determined because of the contamination layer, discrepancies are observed in the height measurement.

### Development of a specific transfer standard dedicated to AFM/SEM measurement

The calibration of the instruments is a determining step in the measuring process to provide traceable and comparable measurements. To calibrate both AFM and SEM used in this study, specific gratings were developed in collaboration with CNRS/C2N (Centre for Nanoscience and Nanotechnology). These gratings, called P_900_H_60_, consist of a pitch of 900 nm and a step height of 60 nm. The fabrication of these gratings was carried out on silicon wafers. The technique is based on using a direct-writing system (Raith-Vistec EBPG 5000+ electron-beam lithography system) and PMMA resist. After developing, the mask is transferred using RIE (reactive-ion etching).

**Figure 2:**
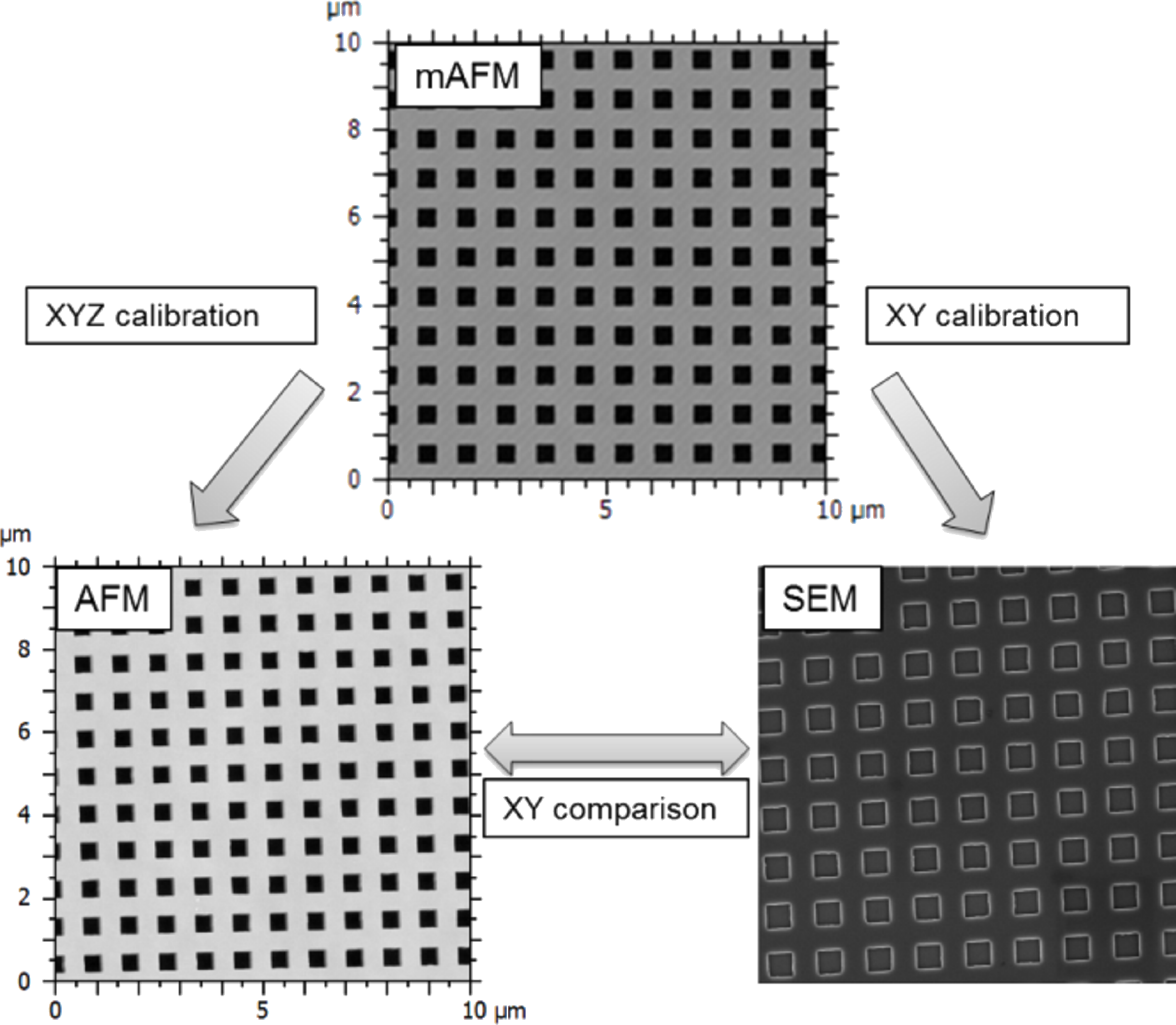
Principle of SEM and AFM calibration using the reference structure measured by mAFM.

The P_900_H_60_ grating is used as a transfer standard and was calibrated by means of the metrological AFM of LNE [12]. This device is a reference instrument specifically designed to establish the traceability route dimensional measurements at the nanoscale making a direct link between the SI meter definition and AFM and SEM measurements. On this instrument, the tip/sample relative position is measured in real time by laser interferometry and the uncertainties associated with the measurements are established through an intensive metrological qualification and modelling of the instrument [20]. The pitch and step height values of the grating resulting from the metrological AFM calibration were found to be (900.17 ± 2.0) nm (*k* = 2) and (53.03 ± 1.0) nm (*k* = 2), respectively. These values had been chosen when the grating was designed to be consistent with the typical range of displacement used on a AFM scanner when imaging nanoparticles (typically 5 µm × 5 µm) and the magnification used on SEM (typically 100000×). Once calibrated, this grating is used in turn to calibrate AFM and SEM (cf. Figure 2). The grating is first measured by AFM and then by SEM to ensure that AFM measurements are not biased by contaminations deposited during e-beam scanning. As the grating is etched on an area of 250 µm, it is difficult to measure exactly at the same place with both instruments. Consequently, five images were acquired by each instrument on different areas of the grating. The mean pitch was evaluated by fast Fourier transform of the image and the obtained results are reported in Table 1.

**Table 1:** Mean pitch measured by mAFM, AFM and SEM along on the grating etched by C2N (P_900_H_60_).

	mAFM of LNE	Veeco AFM before calibration	Zeiss SEM	Veeco AFM after measurements

pitch (nm)	900.17	899.39	902.03	901.61
step height (nm)	53.03	53.65	—	53.26
standard deviation of pitch (nm)	—	0.81	0.61	2.81
standard deviation of step height (nm)	—	0.09	—	0.15
expanded uncertainty for pitch (nm) (*k* = 2)	2	—	—	—
expanded uncertainty for step height (nm) (*k* = 2)	1	—	—	—

The same measurements have been done after measurements performed on nanoparticle populations to ensure that the calibration was still valid and that the instruments have not drifted. The results presented in Table 1 show that the average step height difference from the certified value is covered by the expanded uncertainty (2.0 nm, *k* = 2) of the standard justifying that the NP measurements were made on a calibrated device.

### Development of a repositioning system

Because no instrument is suitable for measuring nano-objects in 3D at the nanoscale, the combination of several techniques is required for completely describing the NP morphology. In this study, we propose to combine two complementary microscopy techniques. However, using this complementarity requires tools capable of locating nano-objects on a substrate and identifying an area of interest. Some solutions are emerging such as the implementation of several instruments within the same chamber or complex algorithms for repositioning with object recognition [21]. The first solution has several advantages, especially, the fact that the different measurements can be performed simultaneously and under the same environmental conditions. This can significantly reduce the measurement discrepancy between techniques. Nevertheless, such an experimental setup is often very expensive and some techniques are not compatible to be embedded within the same chamber.

**Figure 3:**
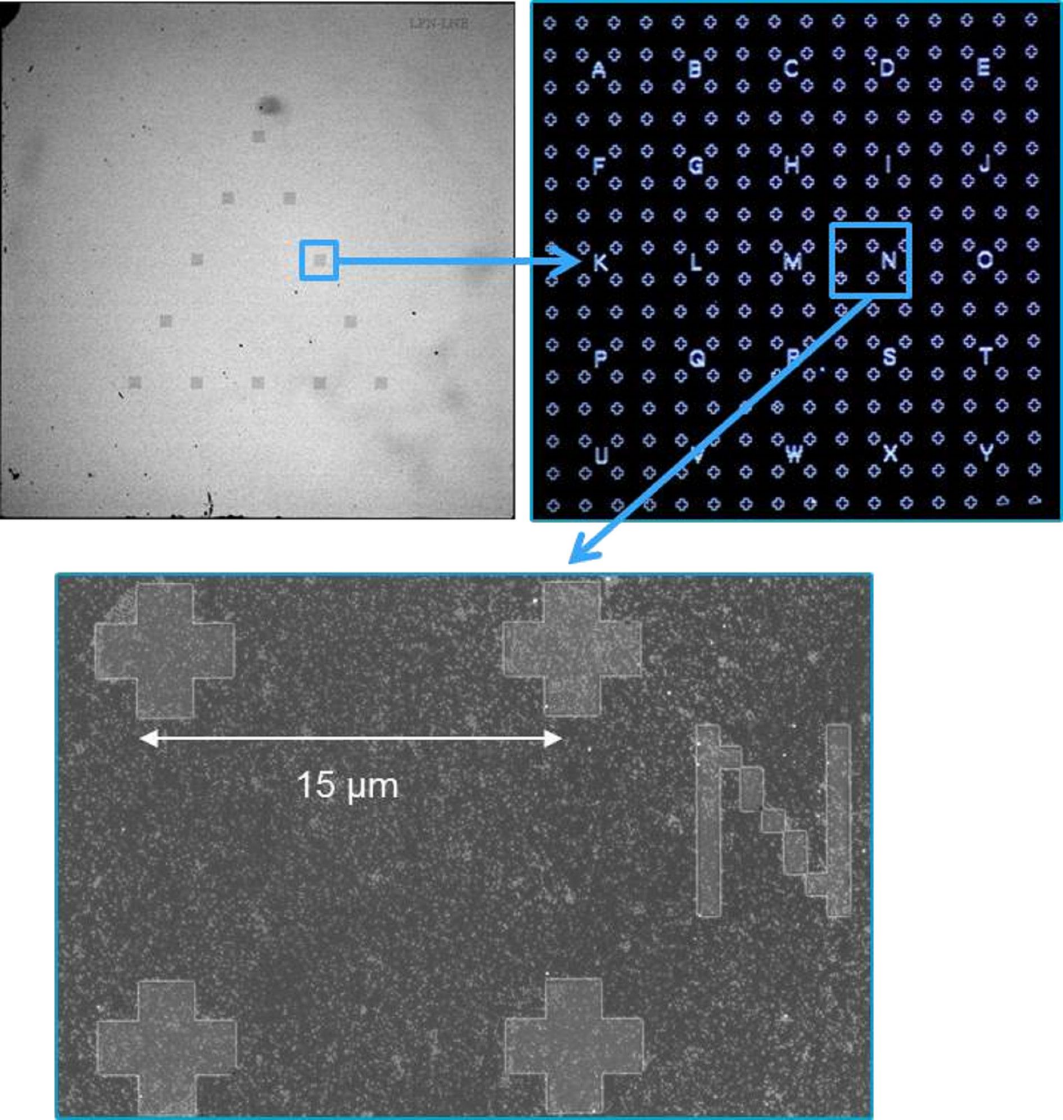
Micro-lithographed chips specifically developed for hybrid metrology and used as a repositioning system.

In this study, we propose to develop a specific lithographed chip with location marks (crosses and letters) compatible with AFM and SEM. These chips were produced in collaboration with the nanofabrication laboratory CNRS/C2N. They enable the quick identification of an area of interest with a set of NPs to be measured with both techniques. The marks are etched in silicon wafer, so the surface is particularly suitable for a controlled deposition of NPs implementing the spin-coater detailed in [11]. The best coverage of isolated NPs has been found in an intermediary area between the substrate edges and the central area very close to rotation axis of the spin-coater. As a consequence, the patterns composed of localization marks were localized in this intermediary area (Figure 3). Twelve patterns were etched forming a triangle. This triangular shape with only one side parallel to the substrate edge allows us to easily orientate the sample on the AFM stage.

The areas of interest consist of squares formed by four crosses with a letter (from A to Y) nearby (Figure 3) for an easy localization of NPs to be analyzed. The distance between two crosses is 15 µm to allow the AFM to scan a complete square on a single image. As detailed above, the production method is based on lithography using a direct-writing system and a PMMA resin in which various patterns are then made by reactive ion etching (RIE). Some tests were also carried out with lift-off techniques for metallic deposition, but the results were found to be less conclusive for the deposit.

## Results and Discussion

### Repeatability of AFM and SEM measurements

The repositioning system allowed us to evaluate the type-A uncertainty (statistical analysis) by estimating the repeatability of both measuring methods. By definition, the repeatability assesses the agreement between the results of successive measurements of the same measurand carried out under the same measurement conditions (same protocol, same operator, same measuring instrument used under the same conditions, repetition over a short period of time). The evaluation of the repeatability of the measurements on the same population requires to restart the measuring process from the initial instrument state (off-state) and to reach the exact location of this population. The SEM high voltage was switched off between each measurement and the AFM tip was withdrawn. The same sets of 50 NPs and 116 NPs of FD304 were measured consecutively four times by AFM and SEM, respectively. For both techniques, four images were recorded with the same adjustment parameters. Of the 50 NPs imaged four times by AFM, the mean height is 25.4 nm and the type-A uncertainty has been estimated to be 0.4 nm. The same statistical analysis was carried out on the 116 NPs imaged by SEM and the lateral diameter was found to be 26.4 nm with an estimate of the type-A uncertainty of 0.5 nm.

### Direct comparison of AFM and SEM measurements on the same set of FD304 nanoparticles

**Figure 4:**
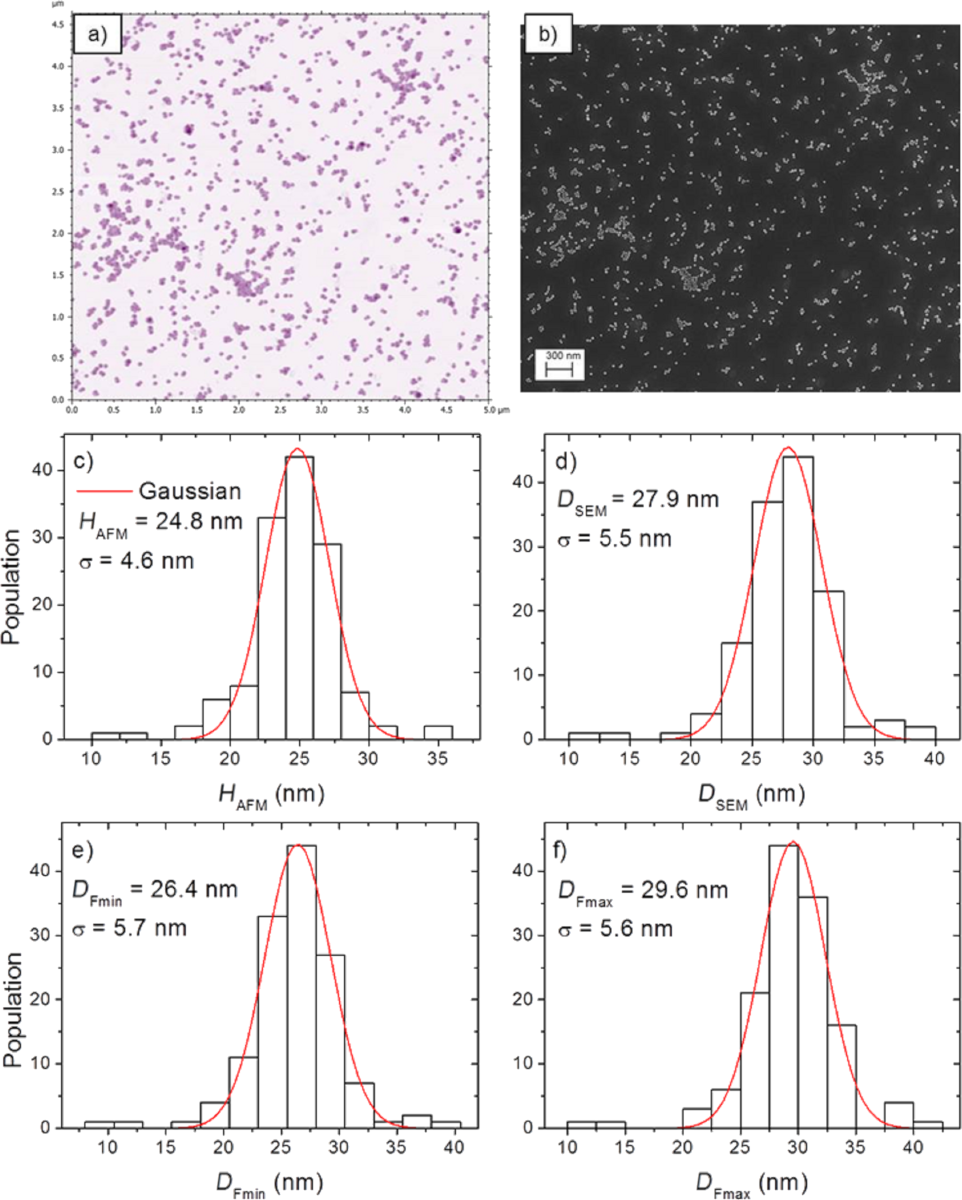
Silica FD304 NPs deposited on the repositioning chip for a direct comparison of a) AFM and b) SEM measurements on the same set of 136 nanoparticles. Histograms of size distribution of FD304 NPs created from AFM and SEM measurements: (c) *H*_AFM_, (d) *D*_SEM_, (e) *D*_Fmin_, (f) *D*_Fmax_.

The colloidal suspension of FD304 NPs was deposited on a silicon wafer by following the protocol detailed in section “Materials and methods”. An area of interest was identified and imaged by AFM (Figure 4a) and SEM (Figure 4b). From these measurements, the size distribution histograms of both techniques were created and are given in Figure 4c–f. The modal values of *H*_AFM_, *D*_SEM_, *D*_Fmin_ and *D*_Fmax_ measured on these 136 NPs have been found to be 24.8 nm, 27.9 nm, 26.4 nm and 29.6 nm, respectively. Discrepancies are thus observed between the NP heights measured by AFM and their SEM lateral diameters. This must be compared with the results of uncertainty budgets previously established for measuring nanoparticles by AFM (*H*_AFM_) [1] and SEM (*D*_SEM_) [22]. In these studies, the main error sources as well as the effect of various imaging parameters on the measurements of the NP height by AFM and the equivalent diameters by SEM have been evaluated. In both cases, a major contribution originates from the calibrating process and uncertainties related to standards. In this new study, the use of our new P_900_H_60_ standard and the implementation of mAFM have allowed us to establish a common traceability chain for both instruments and to reduce the measurement uncertainties to 0.6 nm (*k* = 1) and 2.0 nm (*k* = 1), for AFM and SEM, respectively.

The data provided in the FD304 certificate are based on the size distribution histogram of NP population measurements performed by electron microscopy (SEM and TEM). Consequently, the SEM area-equivalent modal diameter (modal *D*_SEM_ = 27.9 nm) appears to be closer to the FD304 calibration certificate (compared with *H*_AFM_ = 24.8 nm), giving an indicative value of the modal diameter obtained by SEM and TEM equal to 27.8 ± 1.5 nm (*k* = 1) [16]. Other NP populations were analysed on the same sample and similar results were found. As observed in another study, a systematic discrepancy between AFM and SEM has been measured with systematically lower values obtained during the AFM height measurements [23].

### Investigation performed on silica nanoparticles over the whole nanoscale range

In order to further investigate the discrepancy observed between AFM and SEM measurements, various nanosilica populations were measured with mean sizes ranging from 10 to 110 nm. The studied samples consisting of suspensions of nanoparticles with a similar chemical composition but of different origins (reference materials, commercial products or synthesized samples) are listed in Table 2 and have been deposited on repositioning systems described in section “Development of a repositioning system” following the protocol outlined in section “Materials and method”. A total of 1050 NPs was analyzed in such a way that the results are comparable and statistically representative of the NP populations (Table 2).

**Table 2:** Area equivalent modal diameter, measured by SEM and number of NP analyzed for each population used in this study.

NPs	*D*_SEM_ (nm)	*D*_SEM_ reference value (nm)	number of NPs analysed

FD304	27.9	27.8 (indicative value)	132
FD102 1st mode	18.4	18.2	166
Klebosol 30R50 1st mode	37.6	—	235
Klebosol 30R50 2nd mode	80.1	—	313
OT R3	104.8	—	204

**Figure 5:**
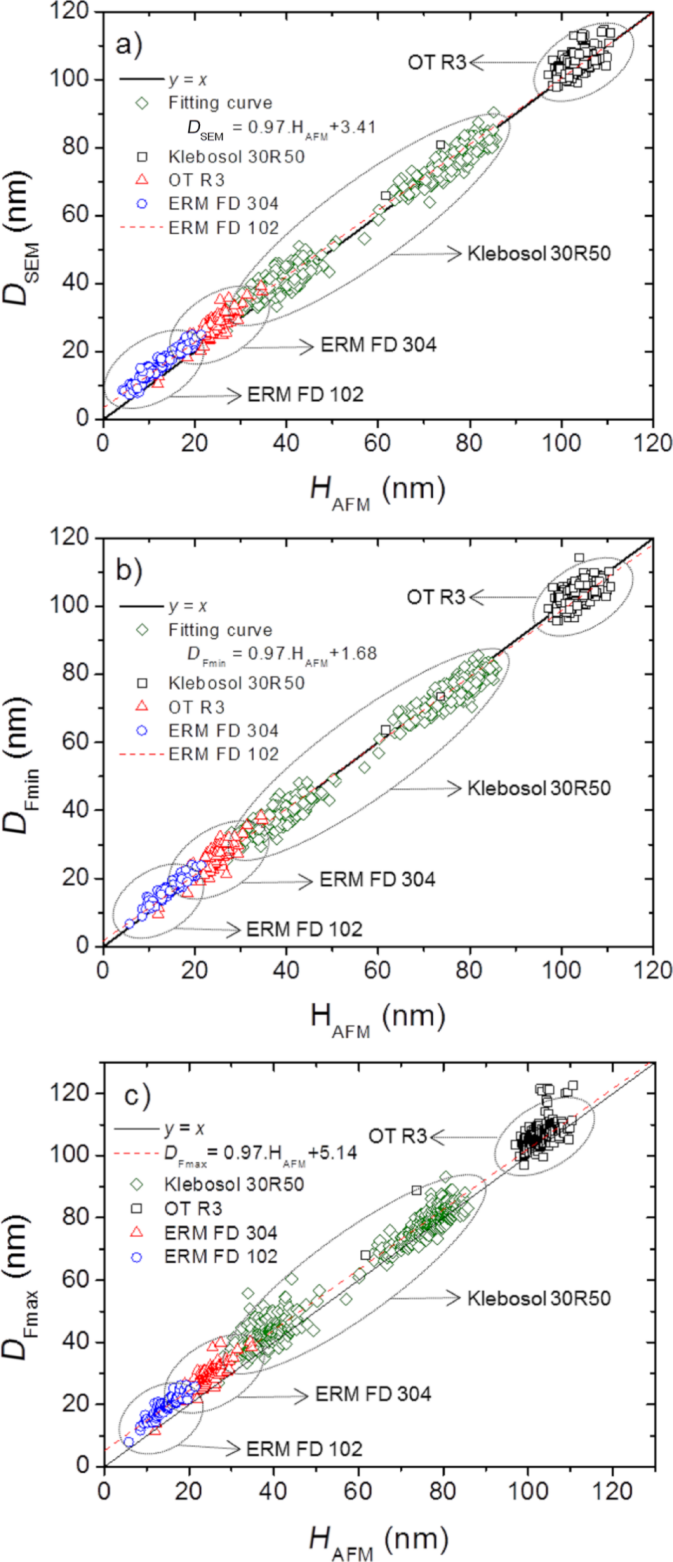
Comparison of NP height measurements performed by AFM and: (a) *D_SEM_*, (b) *D*_Fmin_, (c) *D*_Fmax_ of several silica NP populations.

All results of the measurements are reported in Figure 5 in three graphs showing *D*_SEM_, *D*_Fmin_ and *D*_Fmax_ as a function of the AFM height, *H*_AFM_. This figure displays that the size of various NP populations (for a total of 1050 NPs) ranges from 10 to 110 nm. The curve *y* = *x* was added to highlight the eventual discrepancies between AFM and both SEM measurements. Firstly, we can notice that the whole measurements follow a linear law different from *y* = *x*, of which the equation is given on the graph. The results follow linear laws expressed by the relationships *D*_SEM_ = 0.97·*H*_AFM_ + 3.41 (coefficient of determination *R*^2^ = 0.99), *D*_Fmin_ = 0.97·*H*_AFM_ + 1.68 (*R*^2^ = 0.99) and *D*_Fmax_ = 0.97·*H*_AFMmm_ + 5.14 (*R*^2^ = 0.99). There is no noticeable break of behavior among the samples inducing that the relationship between height and lateral diameters does not depend on the origin of the sample. Moreover, the dispersion of measurements seems to decrease with the NP size. So, this dispersion is not linked to measurement uncertainties, which should be larger for smaller NP because of measuring difficulties. Indeed, for FD102 reference particles, the dispersion peak-to-peak has been found to be close to ±2 nm, and ±5 nm for OT R3 particles. This observation will be discussed below in section “Influence of deviation from sphericity”.

In the graph showing *D*_Fmin_ as a function of *H*_AFM_ (Figure 5b) the fitted curve (dashed line) is very close to the curve *y* = *x*, but the majority of measurements of nanoparticles smaller than 40 nm lie above. This means that the NP heights measured are lower than the SEM lateral diameters. However, regarding the uncertainties linked to both instruments (0.6 nm (*k* = 1) and 2.0 nm (*k* = 1), for AFM and SEM, respectively), these differences are not statistically significant.

In contrast, the discrepancies between *H*_AFM_ and *D*_Fmax_ are more pronounced yielding an aspect ratio different from 1 for all investigated nanosilica populations. The measured AFM height is generally lower than the SEM diameters. This rises the following questions: Is the particle deformed on the substrate due to capillary forces? Does the measured discrepancy between AFM and SEM depend on the chemical composition?

### Influence of the NP chemical composition on the AFM/SEM measurements

In order to determine the influence of the physico-chemical properties of the nanomaterial on AFM and SEM measurements, NPs with very different chemical compositions and mechanical properties compared to nanosilica were imaged by both techniques. A polystyrene latex (PSL) NP population was chosen because the NPs are well-known for having a spherical shape [24]. The PSL sample used in this study has a modal diameter close to 30 nm and can be thus directly compared with the FD304 NP population.

**Figure 6:**
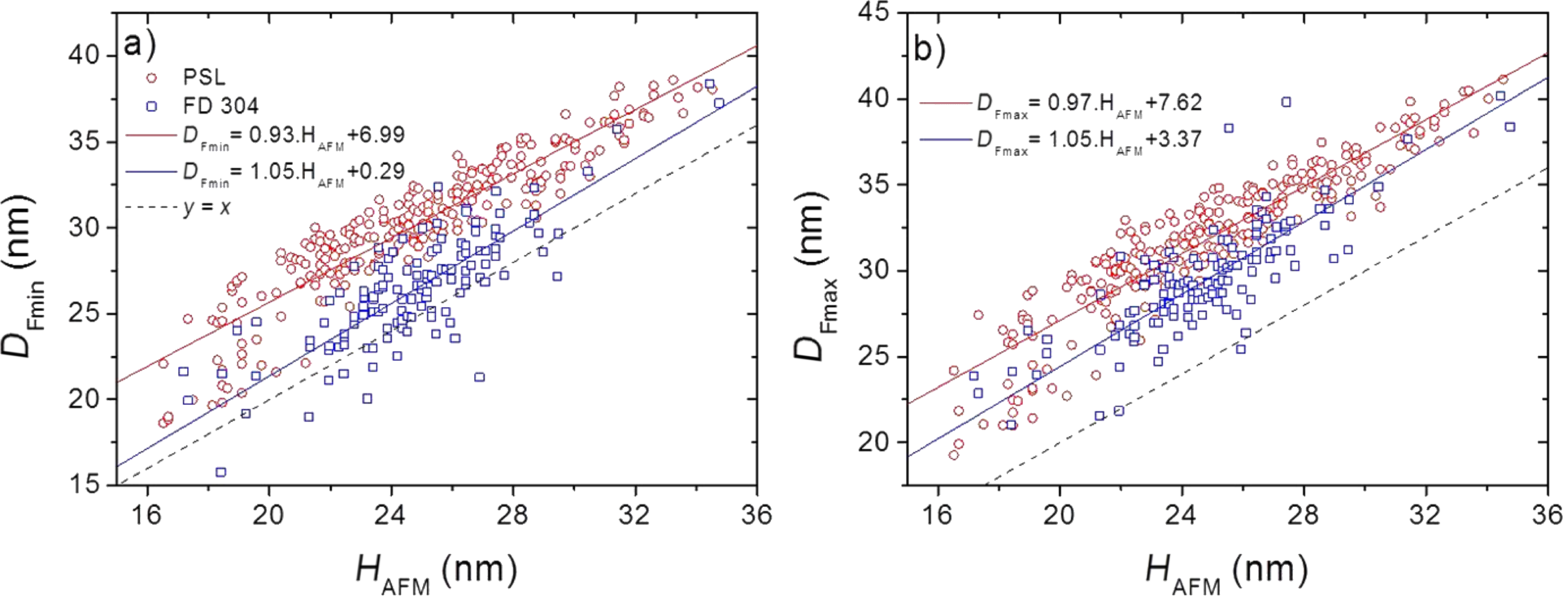
Comparison of AFM height measurement, *H*_AFM_ and (a) *D*_Fmin_, (b) *D*_Fmax_ on the same sets of PSL and SiO_2_ NPs.

Measurements were performed using AFM and SEM on the same set of 257 PSL NPs. Concerning SEM, the imaging parameters detailed in section “Investigation performed on silica nanoparticles over the whole nanoscale range” have been used. The comparison of size measurements of FD304 NPs and PSL NPs using both microscopy techniques is given in Figure 6. Regardless of the nature of the sample, the behavior is similar and a systematic discrepancy is observed between the diameters measured with SEM and the heights measured with AFM. However, the systematic deviation between SEM and AFM measurements is 3 nm larger for PSL NPs.

With regard to the values of *D*_Fmin_ and *D*_Fmax_ the linear fits follow the equations *D*_Fmin_ = 1.05·*H*_AFM_ + 0.29 and *D*_Fmax_ = 1.05·*H*_AFM_ + 3.37 for silica nanoparticles. This indicates that, on average, a discrepancy of 3 nm is observed between *D*_Fmin_ and *D*_Fmax_. Moreover, this discrepancy is larger than the uncertainties linked to both instruments. In contrast, for PSL particles with diameters in the same range, the linear fits are similar, *D*_Fmin_ = 0.93·*H*_AFM_ + 6.99 and *D*_Fmax_ = 0.97·*H*_AFM_ + 7.62, demonstrating that *D*_Fmin_ and *D*_Fmax_ are very close.

As a conclusion, the information can be summarized as follows (within the uncertainties):

- silica nanoparticles: (*D*_Fmin_ ≈ *H*_AFM_) < *D*_Fmax_
- PSL nanoparticles: (*D*_Fmin_ ≈ *D*_Fmax_) > *H*_AFM_

**Figure 7:**
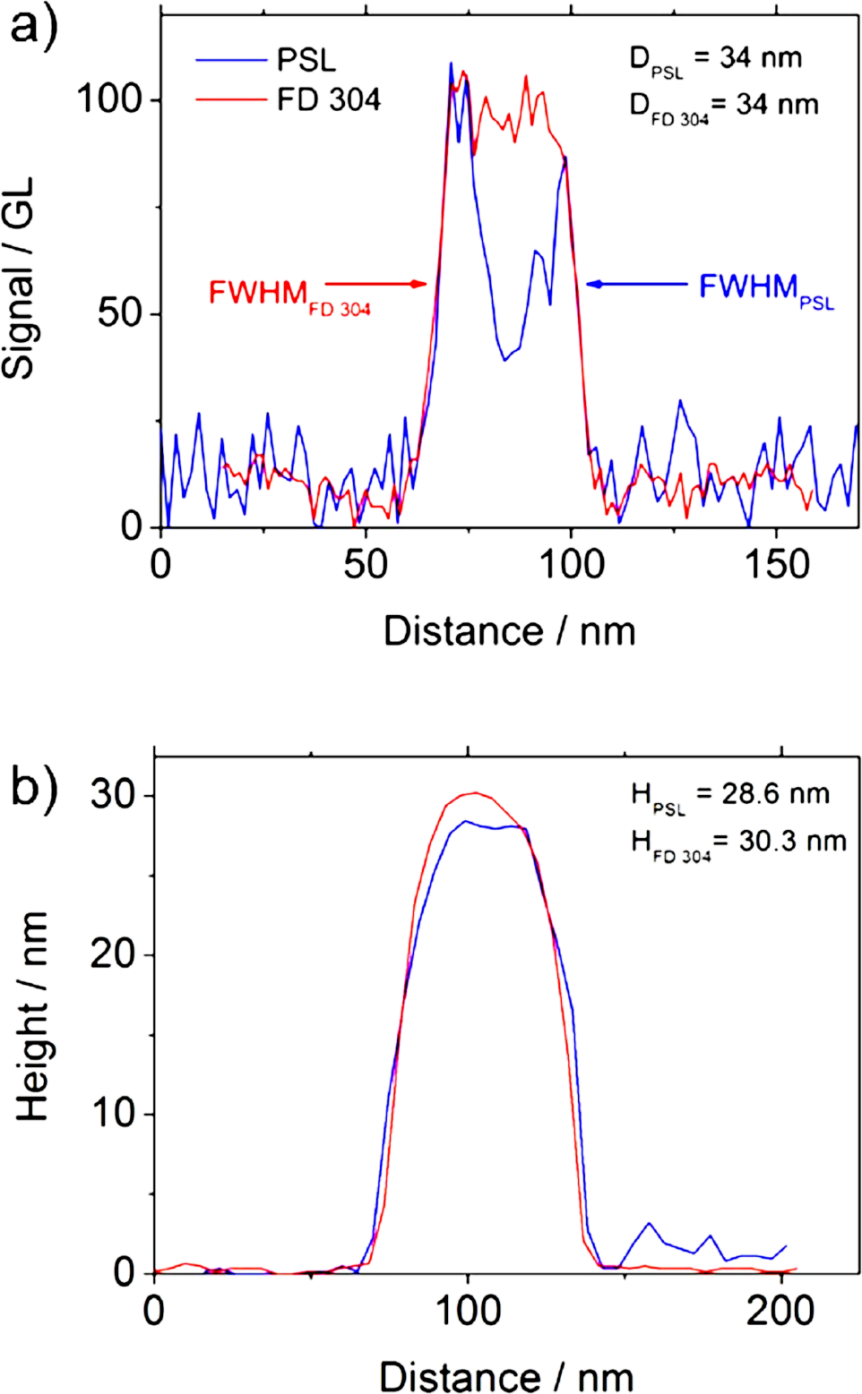
Profiles obtained by (a) SEM and (b) AFM, on a single NP of FD304 or PSL with the same diameter measured by SEM.

**Figure 8:**
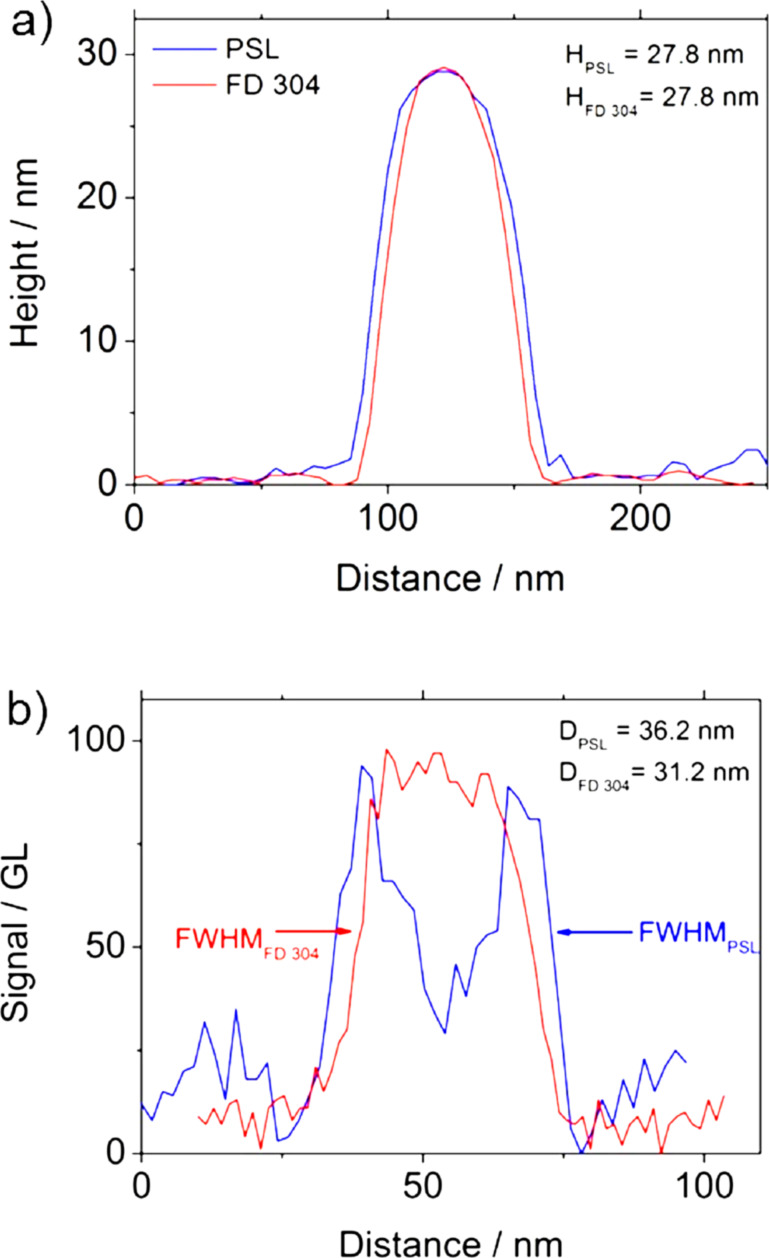
Profiles obtained by (a) AFM and (b) SEM, on a single NP of FD304 or PSL with the same height measured by AFM.

In order to ensure that the measured difference is not due to image analysis processing, the profiles of PSL and FD304 NPs have been measured and are presented in Figure 7 and Figure 8. The profiles correspond to the intensity in grey level and the topography of the sample along the NP diameter for SEM and AFM measurements, respectively. For simplicity, the SEM diameter used here corresponds to *D*_SEM_, i.e., the projected area-equivalent diameter.

Figure 7a and Figure 7b represent the profiles obtained by SEM and AFM respectively, on two single NPs of FD304 and PSL. In this case, although the shape of the SEM profiles are different, the diameter measurements give the same result (*D*_PSL_ = *D*_FD304_ = 34 nm). However, a discrepancy to the AFM measurements equal to 1.7 nm is observed. Figure 8a and Figure 8b give an opposite example showing two single NPs of PSL and silica with the same AFM height (*H*_PSL_ = *H*_FD304_ = 27.8 nm) and a discrepancy of 5 nm on the diameter measurement. The AFM measurements on PSL and FD304 NPs were carried out by using a different tip. Consequently, the measurements of the lateral diameter in the AFM profiles are not comparable.

Figure 7 and Figure 8 show that the SEM profiles of the PSL nanoparticle have an M-shape at 3 kV and are very different from the silica signal. However, the lateral diameter measurements performed at FWHM are similar for both kinds of NP. Moreover, these measurements have been carried out by maintaining the adjustment parameters constant, that is, with the same dimensional properties of the electron beam. Consequently, the difference in behavior observed between AFM and SEM measurements performed on PSL and silica NPs are not due to image analysis processing but may come from other phenomena discussed below.

### Possible origin of the observed discrepancies

From the results detailed above, several hypotheses have been discussed regarding the discrepancy observed between AFM and SEM measurements. Here, the main question is to know if this discrepancy may be explained by either nanoparticle deformation or a non-spherical shape of nanoparticles.

### Nanoparticle deformation

In previous studies, comparable differences between measured height and diameter of NPs have been observed already. For instance, several studies show that PSL NPs could be squished on the substrate causing a deformation of the NP due to capillary forces [25–26]. Indeed, for a PSL NP with a nominal diameter equal to 50 nm, the difference between height and diameter measured by electron tomography is 6.5 nm, even reaching 7.4 nm for a 100 nm nominal diameter. A quantitative model indicates that the difference between diameter and height of a 30 nm PSL NP is approximately 17%, i.e., 5.1 nm. These theoretical values are slightly lower than the ones experimentally observed. Furthermore, other studies focused on the possible deformation of PSL NPs due to the interaction with the AFM tip during scanning [27–28]. Their results show a possible deformation of the NP under the tip, demonstrated by measurements performed using the peak-force tapping AFM mode. This hypothesis is supported by the relationship (*D*_Fmin_ ≈ *D*_Fmax_) > *H*_AFM_ deduced from Figure 6 in section “Influence of the NP chemical composition on the AFM/SEM measurements ” regarding the PSL measurements. This demonstrates that the PSL nanoparticle is compressed by the tip with a uniform force applied in the *XY*-plane.

The question is whether the silica NPs can be distorted by an interaction with the substrate in a way similar to the PSL NPs. However, the mechanical properties of silica are different than those of PSL even at the nanoscale. The Young’s modulus of PSL NPs has been found to be equal to 8.0 GPa for 60 nm particles [29]. In comparison, the Young’s modulus of silica NPs with similar size was evaluated at 68.9 ± 9.6 GPa [30]. This value is consistent with the bulk value (72 GPa) [31].

The Hertz theory [32] can describe the elastic contact of a sphere of radius *R* with a half space. Under an applied force *P*, the elastic deformation leads to a circular contact area of radius *a*_H_ defined by:

[1]
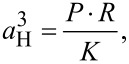


where *K* the equivalent elastic modulus of the NP and the substrate defined by:

[2]
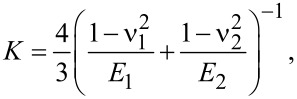


where *E*_i_ and *ν*_i_ are the Young’s modulus and the Poisson’s ratio of the sphere and the half space. The depth of indentation δ, i.e., the elastic displacement is defined by:

[3]
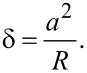


In the case of silica NPs deposited on a Si substrate under ambient conditions, since the area/volume ratio becomes important, capillary adhesion forces must be taken into account. Deformations of the silica NP and substrate can be assessed using the model developed by Derjaguin, Muller and Toporov (DMT) [33]. This model describes the elastic deformation of spherical bodies by including adhesion forces to the Hertz contact equation. This interaction can be described by the Bradley theory [34]:

[4]
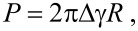


with *P* the adhesion force and Δγ = γ_1_ + γ_2_ − γ_12_ the work of adhesion with γ_1_ and γ_2_, the surface energies of NP and substrate, respectively, and γ_12_ the interfacial energy.

Adding this force to the Hertz model described in Equation 1, as there is no other external force applied on the system, the contact radius *a*_DMT_ between the NP and the substrate regarding DMT model can be determined using the relation:

[5]
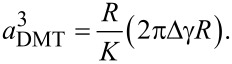


The whole deformation of the system δ can be then determined using the equation:

[6]
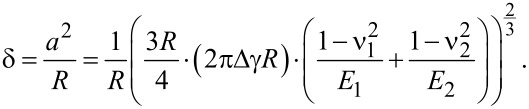


In this study, silica NPs are deposited on silicon wafer with (111) orientation. The corresponding values of these materials are detailed in Table 3.

**Table 3:** Parameter values used for the deformation calculation.

parameter	value	reference

γ_Si(111)_	1.24 J/m²	[35]
γ_SiO2_	0.259 J/m²	[36]
*E*_Si(111)_	160 GPa	[37]
*E*_SiO2_	68.9 GPa	[30]
ν_Si(111)_	0.27	[37]
ν_SiO2_	0.18	[38]

As explained in [39], the uncertainty of δ can be expressed as a rectangular distribution of the half-width δ/2 and is equal to:

[7]
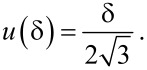


Finally, the theoretical deformation of the system is equal to 0.6 ± 0.2 nm for a 30 nm nominal diameter silica NP, 0.8 ± 0.3 nm for a 100 nm silica NP. Consequently, the 3.5 nm discrepancy between AFM and SEM measurements for FD304 particles cannot be fully explained by NP deformation due to adhesion forces.

### Influence of deviation from sphericity

An additional effect must be taken into account to explain the difference between the two measurements. Even if silica NPs are well-known for having a spherical shape, a systematic control of the sphericity of each NP population used in this study is necessary. Indeed, a deviation from sphericity in shape could induce discrepancies between AFM height and SEM lateral diameter measurements. For instance, a study of the aspect ratio of the two modes of ERM-FD102 was carried out during the certification process of the NPs by electron microscopy [15]. According to the certification document, the particle aspect ratio is defined as the ratio of the major diameter (length) to the minor diameter (width) of a fitted ellipse and has been found to be 1.1 and 1.0, respectively, for the first and second class. Because of the tip/NP convolution and the consequences on the AFM measurements, it is impossible to obtain reliable information on the shape of the NPs in the three spatial directions with such a technique. An estimate of the NP shape can be obtained only by analysing SEM images assuming symmetry along the major axis.

**Figure 9:**
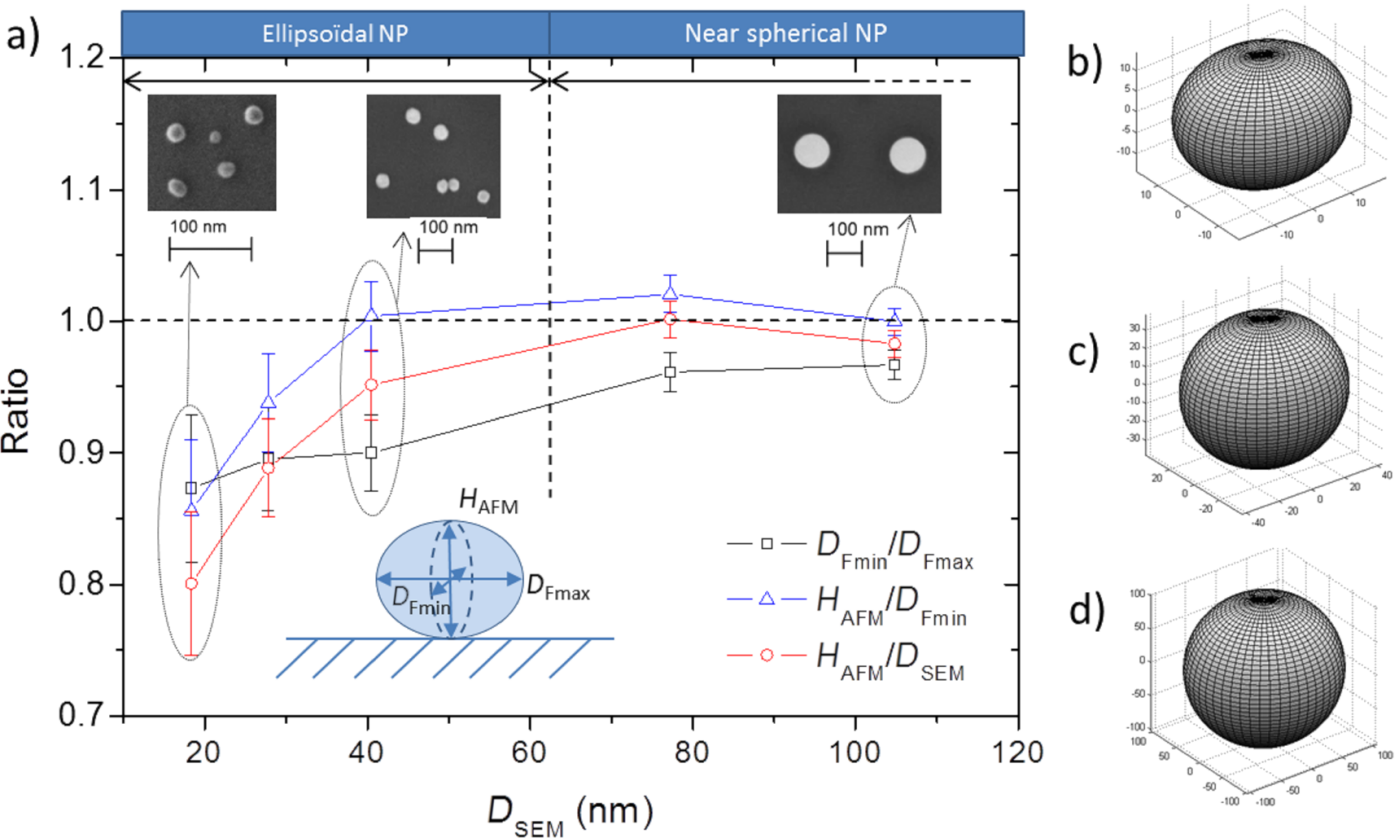
(a) Comparison of (*D*_Fmin_/*D*_Fmax_), (*H*_AFM_/*D*_Fmin_) and (*H*_AFM_/*D*_SEM_) for each NP population (ERM-FD102, ERM-FD304, Klebosol 30R50 1st mode, Klebosol 30R50 2nd mode and OT R3). MATLAB modelling of the NP shape of (b) FD102, (c) 1st mode of 30R50 and (d) OTR3 based on the average values of *D*_Fmin_, *D*_Fmax_, and *H*_AFM_. The *D*_SEM_ values (projected area-equivalent diameter) are listed in Table 2.

From measurements performed in section “Investigation performed on silica nanoparticles over the whole nanoscale range”, the aspect ratio (*D*_Fmin_/*D*_Fmax_) has been calculated for each NP (for a total of 1050 NPs). The mean aspect ratio and the corresponding standard deviation have been also determined for each population (FD102, FD304, 30R50 1st and 2nd mode and OTR3). The same study has been performed for (*H*_AFM_/*D*_Fmin_) and (*H*_AFM_/*D*_SEM_). The results are presented in Figure 9a. The bars represent the sphericity distribution (standard deviation) associated with each NP population. We can notice that distribution decreases when the NP size increases, proving the shape distribution is more significant for the smaller nanoparticles.

The result obtained from the FD102 population, 0.87 ± 0.08, is in accordance with the calibration certificate (minor over major diameter of the ellipse equal to 0.9, corresponding to 1.1 in the certificate). These results show that sphericity of the silica NPs increases with size. Indeed, the average aspect ratio (*D*_Fmin_/*D*_Fmax_) of FD304 particles (*D*_SEM_ = 27.8 nm), has been found to be equal to 0.90 ± 0.04, while the mean aspect ratio of OTR3 particles (*D*_SEM_ = 104.8 nm), is equal to 0.97 ± 0.01. Moreover, the ratio (*H*_AFM_/*D*_Fmin_) is equal to 0.94 ± 0.04 for FD304 NPs and 1.00 ± 0.01 for OTR3 particles. Moreover, the evolution of the (*D*_Fmin_/*D*_Fmax_) ratio as a function of the NP size is slightly different from that of the (*H*_AFM_/*D*_Fmin_) ratio. From these observations, NPs can be classed in several categories: In the range from 40 to 110 nm, almost equal results of *H*_AFM_ and *D*_Fmin_ measurements are observed, which indicates that the 2nd mode of 30R50 (*D*_SEM_ = 80.1 nm) and the OTR3 (*D*_SEM_ = 104.8 nm) particles are nearly spherical with no preferential orientation on the substrate. The lack of preferential orientation can likely explain the fact that, as shown in Figure 7, the measurement dispersions are higher for near-spherical nanoparticles with sizes larger than 40 nm.

Regarding the 1st mode of the 30R50 population (*D*_SEM_ = 37.6 nm), the ratio (*H*_AFM_/*D*_Fmin_) is equal to 1.00 ± 0.03, suggesting that these NPs have a rotational symmetry along the major axis. However, for these particles, the (*D*_Fmin_/*D*_Fmax_) ratio is equal to 0.90 ± 0.03. This proves that these particles are slightly elongated and lie on the substrate in such a way that their major axis is parallel to the surface. Consequently, their height measurement (*H*_AFM_) becomes comparable to the lateral diameter value measured along the minor axis (*D*_Fmin_).

Finally, for particles with a nominal diameter under 30 nm (FD102 and FD304), the (*H*_AFM_/*D*_Fmin_) ratio is significantly different from 1. Moreover, the (*D*_Fmin_/*D*_Fmax_) ratio is also noticeably lower than 1. This demonstrated that these NP have an ellipsoidal shape with *D*_Fmax_ > *D*_Fmin_ > *H*_AFM_.

## Conclusion

In this study, a novel approach of hybrid metrology is presented combining AFM and SEM measurements for a metrological dimensional characterization of nanoparticles. To this end, several tools have been developed: specific standards, a custom-written software for image analysis and processing compatible with both microscopy techniques, and a branded repositioning system. This latter makes it possible to directly measure the same set of NPs by using two different microscopy-based techniques (AFM and SEM). These two techniques have been chosen because they give complementary information on the NP size. SEM provides a metrological measurement in the *XY*-plane, while the AFM gives similar information along the *Z*-axis.

The instruments were calibrated using a new specific transfer standard dedicated to AFM/SEM measurements. A reference standard was calibrated thanks to the metrological AFM of LNE, establishing a direct link between the SI meter definition and the NP measurements.

A direct comparison of SEM and AFM measurements has been performed on several silica populations of similar chemical composition but coming from different sources and with dimensions ranging from 10 to 110 nm. Surprisingly, all measurements follow a linear law regardless of the origin of the sample. Furthermore, experimental data progressively deviate from the curve *y* = *x* when the silica NP size decreases. Thus, a systematic discrepancy for the lowest values is obtained between the two measurements with SEM and AFM. A study of silica NP deformation using the DMT model has demonstrated that deformation alone cannot fully explain the observed discrepancy. However, the study of the deformation of silica NPs under an AFM tip should be investigated more intensively.

Consequently, the implementation of the hybrid metrology with the combined use of AFM and SEM allowed us to accurately study the size-dependence of the silica nanoparticle shape. Several categories of nanoparticles were determined. In the range from 40 to 110 nm, silica nanoparticles are nearly spherical; around 40 nm, their shape is elongated with a symmetry axis along the major axis; below 40 nm, the NPs are ellipsoidal without symmetry axis and with *D*_Fmax_ > *D*_Fmin_ > *H*_AFM_.
